# Macular Diplopia Caused by Epiretinal Membrane

**DOI:** 10.7759/cureus.106774

**Published:** 2026-04-10

**Authors:** Joel Gutovitz, Minal Patil, Shneior Z Korf, Einat Shneor, Yolanda Friedrich, Orwa Nasser

**Affiliations:** 1 Ophthalmology, Sheba Medical Center, Ramat Gan, ISR; 2 Ophthalmology, Drishti Eye Institute, Dehradun, IND; 3 Medicine, Rambam Medical Center, Technion - Israel Institute of Technology, Haifa, ISR; 4 Optometry, Jerusalem Multidisciplinary College, Jerusalem, ISR; 5 Ophthalmology, Leumit Health Care Services, Haifa, ISR; 6 Ophthalmology, College of Optometry, University Eye Institute, University of Houston, Houston, USA; 7 Ophthalmology, College of Optometry, Arab American University of Palestine, Ramallah, PSE; 8 Ophthalmology, Orasis Eyecare Center, Ma'alot-Tarshiha, ISR

**Keywords:** central-peripheral rivalry, diplopia, double vision, dragged-fovea diplopia syndrome, epiretinal membrane, esotropia, exotropia, hypertropia, hypotropia, macular diplopia

## Abstract

Macular diplopia, also known as dragged-fovea diplopia syndrome, is a rare form of binocular diplopia resulting from foveal displacement caused by macular pathology. Epiretinal membrane (ERM) is the most common underlying cause, producing retinal misregistration that disrupts central fusion while preserving peripheral correspondence. Recognition of this entity is critical to avoid misdiagnosis as strabismic or neurologic diplopia.

This type of diplopia highlights the sensory nature of macular diplopia, which arises from foveal displacement and central-peripheral fusion conflict. ERM-induced traction alters retinal correspondence, causing diplopia despite normal ocular alignment. Although surgical membrane peeling can correct macular anatomy, diplopia may persist due to limited cortical adaptation. Conservative management using prismatic correction or partial occlusion remains an effective first-line approach.

In conclusion, epiretinal membrane-associated macular diplopia is an uncommon but clinically significant cause of binocular double vision. Accurate diagnosis through multimodal imaging and orthoptic evaluation enables tailored management and can prevent unnecessary surgical or neurological interventions.

## Introduction and background

Macular diplopia, also known as dragged-fovea diplopia syndrome (DFDS), is a form of binocular diplopia that arises from the disruption of normal foveal alignment caused by macular pathology. This condition occurs when foveal displacement or retinal traction leads to misregistration between corresponding retinal points, resulting in double vision and visual distortion [1]. The most frequent underlying cause is an epiretinal membrane (ERM), a fibrocellular proliferation over the internal limiting membrane that exerts tangential traction on the macula, distorting its architecture and photoreceptor alignment [2].

ERM is a common macular disorder associated with aging, with prevalence estimates ranging from 2.6% to 39% in population-based studies [3]. It may be idiopathic or secondary to ocular conditions such as retinal vein occlusion, diabetic retinopathy, uveitis, trauma, or retinal detachment surgery [4]. The formation of ERM involves glial cell proliferation, Müller cell activation, and extracellular matrix remodeling, leading to macular traction and foveal displacement [5].

Clinically, patients with ERM often report reduced central vision, metamorphopsia, and, less commonly, binocular diplopia due to retinal misregistration [6]. The misalignment between the distorted fovea and the unaffected eye causes central-peripheral rivalry (CPR)-type diplopia, wherein peripheral fusion is preserved while central fusion fails [6]. Optical coherence tomography (OCT) has become the gold standard for diagnosis, allowing precise visualization of macular traction and staging of ERM severity [7].

While ERM is the leading cause of macular diplopia, other conditions can produce similar foveal misalignment, including subretinal neovascularization, vitreomacular traction, macular dystrophies, and complications following retinal detachment or macular translocation surgery [8]. Additionally, secondary macular fibrosis can occur following ischemic or inflammatory disorders such as sickle cell retinopathy, which may also result in diplopia [9].

Understanding the relationship between ERM and macular diplopia is critical, as surgical membrane peeling may improve anatomic outcomes but does not consistently resolve diplopia [10]. Thus, early recognition of ERM-induced foveal displacement is vital for accurate diagnosis, patient counseling, and management planning.

## Review

Overview

An ERM is a fibrocellular proliferation that develops along the inner limiting membrane (ILM) of the macula, often resulting in mechanical traction that distorts the macular architecture and alters normal photoreceptor alignment. The ERM consists primarily of proliferating glial cells, fibroblasts, and myofibroblasts embedded within a collagenous matrix, producing tangential tractional forces that can disrupt the foveal contour, leading to decreased visual acuity, metamorphopsia, and, in rare cases, binocular diplopia [5].

The prevalence of ERM varies significantly between populations, ranging from 2.6% to 39%, with a sharp increase after the age of 50 years and peaking in the 70-79-year age group [3]. The formation of idiopathic ERM is commonly attributed to posterior vitreous detachment, which allows glial migration across the ILM. Secondary ERMs can occur after retinal detachment, diabetic retinopathy, uveitis, trauma, vascular occlusion, or intraocular surgery [2].

ERM progression results in superficial retinal folds, increased macular thickness, and a loss of foveal depression, all of which are easily demonstrated on OCT. Tangential traction may ultimately lead to macular edema or lamellar holes in advanced stages [10]. OCT has revolutionized the understanding of ERM by revealing not only retinal distortion but also disruptions in the outer retinal bands, which correlate with metamorphopsia and decreased visual function. Recent imaging innovations such as OCT angiography (OCTA) have demonstrated reduced FAZ circularity and microvascular distortion in symptomatic ERM cases [11].

Macular diplopia

Macular diplopia, often termed DFDS or CPR-type diplopia, refers to binocular double vision that arises from a sensory conflict between the two eyes rather than from an ocular motility disorder. Described and elaborated by Bixenman and Joffe, it refers to a phenomenon in which central and peripheral images fail to fuse because one fovea has been displaced by macular pathology [12].

The condition is most frequently associated with ERM, but it can also occur following vitreomacular traction, retinal wrinkling, subretinal neovascular membranes, macular dystrophies, and macular translocation surgery [13,14]. When one macula becomes distorted, the corresponding retinal point no longer aligns precisely with its counterpart in the fellow eye. As a result, central fusion is lost while peripheral fusion persists, leading to double vision that cannot be corrected with prism or muscle surgery.

In a seminal analysis of 56 patients with ERM, Veverka et al. found that approximately 21% had symptomatic CPR-type diplopia, while another 60% had subclinical retinal misregistration detectable on specialized tests [6]. Diplopia is often vertical because vertical fusional amplitudes are narrower than horizontal, making vertical displacement of the fovea more symptomatic. Horizontal or torsional diplopia may occur in cases where traction is oblique or asymmetrical.

Mechanisms and pathophysiology

The fundamental mechanism of macular diplopia is retinal misregistration, in which displaced foveal points in the two eyes stimulate noncorresponding cortical loci.

Veverka et al. evaluated 50 patients with ERM, including 25 with diplopia and 25 without, to determine the underlying mechanisms of diplopia [6]. Using detailed orthoptic assessment and tests for retinal misregistration, the authors found that CPR-type diplopia due to retinal misregistration was the most common etiology, accounting for 44% (11/25) of diplopic patients. This phenomenon reflects the disruption of normal retinal correspondence due to macular distortion, resulting in a mismatch between central and peripheral fusion signals. Importantly, retinal misregistration represents a sensory cause of diplopia distinct from ocular misalignment [6]. Despite this, diplopia in ERM was frequently multifactorial. Strabismus was identified in 28% (7/25), while mixed etiologies and refractive causes were also observed. Notably, 60% (15/25) of nondiplopic patients demonstrated retinal misregistration, indicating that this finding alone does not necessarily result in symptomatic diplopia and may be compensated for by sensory adaptation. These results emphasize that clinicians should not attribute diplopia solely to retinal pathology in ERM, as treatable causes such as strabismus or refractive error may be present and represent the primary barrier to single vision [6].

Pehere et al. published a case report describing the management of a patient with binocular diplopia (macular diplopia) secondary to sickle cell retinopathy and associated ERM formation [9]. The report highlights the complex sensory mechanism underlying macular diplopia, in which retinal distortion leads to disruption of central fusion despite preserved peripheral fusion. In this patient, the diplopia was attributed to a small-angle deviation in the context of retinal misregistration, making management particularly challenging, especially in a resource-limited setting where advanced diagnostic and therapeutic options may be restricted [9].

Bixenman and Joffe proposed a pathophysiological mechanism involving competition between central and peripheral fusional processes, driven by mechanical distortion of the macula [12]. This CPR disrupts normal binocular integration, rendering standard optical corrections such as prisms ineffective. These findings are particularly important as they delineate a sensory form of diplopia distinct from purely motor causes, emphasizing that retinal pathology can fundamentally alter fusion dynamics. Consequently, management of such patients requires recognition of this mechanism, as conventional strabismus treatments alone may be insufficient without addressing the underlying retinal contribution [12].

Displaced foveal points in both eyes stimulate noncorresponding cortical loci, causing a sensory mismatch that produces rivalrous fusion, a situation in which the brain receives conflicting visual information from each eye [15]. When peripheral images are aligned but central images are discordant, the brain perceives two visual directions for a single object, resulting in diplopia.

Aniseikonia, or unequal perceived image size between eyes, can exacerbate this condition. Retinal stretching caused by ERM-induced traction can alter photoreceptor spacing, producing micropsia or macropsia. This difference in image scale hinders binocular fusion [16].

Hatt et al. evaluated 124 patients with ERM who reported monocular eye closure and compared them with 11 control patients without eye closure to investigate the mechanisms underlying this behavior [17]. The authors identified three principal associations: binocular interference in 29% (36/124), central-peripheral rivalry (CPR)-type diplopia in 27% (34/124), and other causes, primarily strabismus, in 44% (54/124). Notably, binocular interference was defined as monocular eye closure in the absence of diplopia or strabismus, suggesting a distinct sensory disturbance rather than a classic binocular misalignment. This study expands the spectrum of binocular dysfunction in ERM beyond diplopia alone [17]. Functionally, patients with binocular interference demonstrated significantly reduced quality of life, particularly in reading and general function domains of the AS-20 questionnaire, compared with controls. Moreover, when compared with CPR-type diplopia, these patients exhibited worse visual acuity in the affected eye and greater interocular visual disparity, implicating asymmetric retinal image quality as a key driver of symptoms. These findings support the concept that binocular interference represents a separate clinical entity, likely mediated by cortical suppression or sensory imbalance, and should be specifically recognized during evaluation, as it carries meaningful functional consequences despite the absence of overt diplopia [17]. In other words, while some patients adapt through suppression or by closing one eye, others experience persistent diplopia due to inadequate cortical compensation [17]. Neuroimaging studies suggest that this is due to persistent asynchronous activation in binocular cortical regions even after successful anatomical correction [18].

Foroozan and Arnold described one patient (one affected eye predominantly), a 69-year-old man who developed binocular vertical diplopia following bilateral cataract surgery [19]. Despite minimal ocular misalignment (two-prism diopter right hypertropia) and normal extraocular motility, the diplopia was not correctable with prism, suggesting a nonmotor etiology. Fundoscopic evaluation revealed an ERM more pronounced in the left eye, accompanied by progressive metamorphopsia and reduced visual acuity. This case illustrates that even small, comitant deviations may become symptomatic when associated with macular pathology, pointing toward a sensory mechanism of diplopia [19].

Following pars plana vitrectomy (PPV) with ERM peeling, the patient experienced complete resolution of diplopia, supporting the role of retinal misregistration due to foveal displacement as the underlying cause [19]. The authors proposed that disruption of central retinal correspondence leads to central-peripheral fusional rivalry, resulting in persistent diplopia despite minimal strabismus. This report emphasizes that macular pathology should be considered in cases of unexplained diplopia after cataract surgery, particularly when prism therapy fails, and highlights that addressing the retinal abnormality can restore binocular function [19].

Langmann et al. evaluated 264 patients (264 eyes undergoing encircling procedure) for retinal detachment to assess the frequency and characteristics of postoperative diplopia due to motility disorders [20]. At six months, 18 patients (7%) developed diplopia in primary gaze, most commonly associated with esotropia combined with vertical deviation (55%). These findings indicate that diplopia following encircling procedures is relatively uncommon but clinically significant, typically arising from mechanical restriction or altered extraocular muscle function rather than sensory retinal causes [20].

De Pool et al. analyzed 83 patients (95 affected eyes) diagnosed with DFDS over an extended period at a tertiary referral center [21]. This condition was characterized by binocular diplopia unresponsive to prism therapy and not attributable to strabismus, in the setting of macular pathology such as ERM or internal limiting membrane abnormalities. A hallmark finding was the presence of central diplopia with preserved peripheral fusion, reflecting retinal misregistration due to foveal displacement. The authors also introduced the “lights on-off test,” in which diplopia resolved under conditions that eliminated peripheral fusion, confirming the diagnosis; all 69 patients tested demonstrated a positive response [21].

Silverberg et al. reported seven patients (seven affected eyes) with binocular diplopia secondary to various macular pathologies, including ERM, subretinal neovascularization, and central serous retinopathy. All patients presented with constant vertical diplopia associated with small-angle, comitant hyperdeviation and preserved extraocular motility, indicating a sensory rather than motor etiology. Importantly, prism correction and refractive manipulation were ineffective, and sensory testing demonstrated peripheral fusion with reduced stereopsis, consistent with CPR due to macular distortion [22].

Benegas et al. evaluated seven patients (seven affected eyes) with binocular diplopia associated with macular disease, including ERM (six patients) and vitreomacular traction (one patient) [23]. All patients demonstrated clinically significant aniseikonia ranging from 5% to 18%, reflecting image size disparity between the two eyes due to photoreceptor displacement or compression. In most cases (5/7), the image in the affected eye appeared larger, whereas in the other cases, it appeared smaller. Importantly, all patients also had small-angle strabismus but were unable to achieve fusion even when the deviation was neutralized with prisms, indicating that image-size disparity played a critical role in their diplopia [23]. The study highlights aniseikonia as a key sensory mechanism contributing to diplopia and fusion failure in macular disease, which may be misinterpreted as central fusion disruption alone. The coexistence of small-angle strabismus can further complicate diagnosis, potentially leading to inappropriate management. Notably, both optical interventions and retinal surgery showed variable effectiveness, and surgical correction did not reliably resolve aniseikonia. These findings underscore the importance of specifically evaluating image size disparity in patients with macular pathology and diplopia, as addressing aniseikonia may be essential for restoring binocular function [23].

Arnoldi and Reynolds reviewed six patients (six affected eyes) presenting with diplopia that was refractory to prism correction and strabismus surgery, aiming to identify clinical features suggestive of underlying macular pathology [24]. All patients reported vertical diplopia, although only one demonstrated an actual vertical strabismus, highlighting a mismatch between symptoms and motor findings. A distinguishing feature was that diplopia improved in dim illumination, implicating reduced peripheral fusion and supporting a sensory mechanism. Additionally, all patients described monocular visual disturbances, such as illusory image movement, when viewing with the affected eye [24]. The study emphasizes a characteristic clinical profile of diplopia secondary to maculopathy, including comitant vertical diplopia, poor response to prism (eating up prism), and limited fusion capacity. These findings reflect CPR due to retinal distortion, rather than true ocular misalignment. Importantly, such patients may initially present without a known history of retinal disease, making recognition of these features critical for appropriate diagnosis and referral. The authors highlight that careful history-taking and sensory testing can provide key diagnostic clues, even before direct retinal examination confirms the underlying pathology [24].

Diagnosis and imaging

Diagnosing macular diplopia requires integrating fundoscopic, orthoptic, and imaging data. Fundus examination typically reveals macular striae, loss of foveal reflex, and vessel tortuosity. OCT confirms ERM by demonstrating increased macular thickness, loss of foveal depression, and corrugated retinal layers [7].

Specialized orthoptic testing can detect misregistration. The “Lights On-Off Test” developed by Guyton helps distinguish CPR-type diplopia: diplopia appears in light (when central fusion is active) but vanishes in darkness [25]. Additional tests, such as the optotype-frame test or synoptophore central-peripheral fusion slides, can quantify the degree of retinal misregistration [26].

High-resolution OCTA also provides insight into microvascular deformation at the macula and the distortion of the foveal avascular zone (FAZ), which correlates strongly with diplopia severity [11].

Management

There is no single definitive treatment for macular diplopia. Management depends on whether the diplopia is primarily sensory (retinal misregistration) or mechanical (ocular misalignment). Table 1 summarizes reported cases of macular diplopia and their retinal pathology, type of diplopia, and management approach.

**Table 1:** Summary of reported cases of macular diplopia and their retinal pathology, type of diplopia, and management approach ERM: epiretinal membrane; VMT: vitreomacular traction; SRNVM: subretinal neovascular membrane; CSR: central serous retinopathy; RD: retinal detachment; CPR: central-peripheral rivalry; IOL: intraocular lens

Study	Retinal pathology	Type of diplopia	Number of patients	Management
Veverka et al. [6]	ERM	Central-peripheral rivalry	50	ERM peeling ± occlusion
Pehere et al. [9]	Sickle-cell retinopathy	Vertical	1	Strabismus surgery
Bixenman and Joffe [12]	Retinal wrinkling/ERM	Vertical	4	Prism therapy
Hatt et al. [15,17]	ERM	Central-peripheral rivalry	50, 124	ERM peeling ± occlusion
Foroozan and Arnold [19]	ERM (post-cataract surgery)	Vertical	1	ERM peeling surgery with visual recovery
Langmann et al. [20]	Post-retinal detachment surgery	Horizontal/vertical	18	Stepwise management (prism → buckle removal → strabismus surgery)
De Pool et al. [21]	ERM	Vertical/torsional	83	ERM peeling + monocular occlusion
Silverberg et al. [22]	SRNVM, ERM, CSR	Vertical	7	Prism + Bangerter foil
Benegas et al. [23]	ERM/VMT	Vertical/horizontal	7	ERM peeling + prism
Arnoldi and Reynolds [24]	Maculopathy	Vertical	6	Strabismus ± prism
Maddii et al. [27]	ERM	Vertical	2	Orthoptic therapy
Xue et al. [28]	Idiopathic ERM	CPR/aniseikonia-related	42	Nonvitrectomizing vitreous surgery
Fison and Chignell [29]	Post-RD surgery	Vertical/horizontal	15	Prism ± buckle removal ± strabismus surgery
Ismaiel et al. [30]	ERM	Torsional	1	IOL-induced blur

Conservative Approaches

Prism therapy: Bixenman and Joffe reported four patients with retinal wrinkling and ERM formation who presented with a distinct pattern of binocular diplopia characterized by small-angle, comitant hyperdeviations and persistent vertical diplopia [12]. Notably, all patients exhibited unstable single vision that was refractory to conventional prism therapy, highlighting the atypical and treatment-resistant nature of this condition. The clinical presentation suggested that even minimal ocular misalignment, when combined with macular pathology, can result in disproportionately severe and symptomatic diplopia [12].

Prismatic correction is often the first-line treatment, particularly for patients with small-angle deviations. However, prisms frequently fail in CPR-type diplopia because the misalignment is sensory, not geometric [15].

Orthoptic treatment:* *Maddii et al. demonstrated successful use of orthoptic therapy in two ERM-related cases, emphasizing that targeted sensory training may restore comfortable single vision [27]. Maddii et al. described two patients (two eyes with unilateral ERM) presenting with DFDS, a form of central binocular diplopia resulting from foveal displacement [27]. Both patients exhibited diplopia attributable to retinal misregistration, where macular distortion led to a mismatch between central and peripheral fusion. Consistent with prior observations, the diplopia was not amenable to prism correction, underscoring the sensory rather than motor origin of the condition. These cases reinforce the concept that even unilateral macular pathology can significantly disrupt binocular integration [27]. Management in these cases focused on orthoptic strategies tailored to individual visual function, rather than conventional optical correction alone. The authors emphasized the importance of addressing subjective symptoms and optimizing residual binocular function through customized approaches, highlighting that successful outcomes depend on multidisciplinary collaboration between ophthalmologists and orthoptists [27]. These findings support a personalized, function-oriented treatment paradigm for dragged-fovea diplopia, in which therapeutic success relies on adapting to the patient’s sensory adaptations rather than attempting to fully restore normal binocular alignment [27].

Partial occlusion: Partial occlusion strategies using Bangerter foils or translucent tape can suppress central rivalry while preserving peripheral fusion.

De Pool et al. demonstrated that conventional therapeutic approaches, such as prisms and strabismus surgery, were ineffective, underscoring the sensory nature of the disorder [21]. However, partial monocular occlusion using translucent tape (Scotch Satin tape) proved beneficial in a substantial subset, with 46 of 64 patients showing symptomatic improvement. This strategy works by selectively degrading central visual input while preserving peripheral awareness, thereby reducing CPR. The study established dragged-fovea diplopia as a distinct clinical entity with characteristic diagnostic features and highlighted practical, noninvasive management options tailored to its unique pathophysiology [21].

Silverberg et al. showed that prism correction and refractive manipulation were ineffective. Instead, management using partially occlusive Bangerter foils (density 0.4-1.0) applied to the affected eye successfully eliminated diplopia in all patients, although with an average reduction of approximately three lines in visual acuity [22]. Despite this induced blur, peripheral fusion was preserved, allowing stable single vision. Symptoms recurred when the foil was removed or weakened, confirming its therapeutic role. These findings support Bangerter foils as an effective, low-cost, and cosmetically acceptable option for managing refractory diplopia caused by macular pathology, particularly when conventional optical corrections fail [22].

Surgical Approaches

Surgical treatment targets the underlying structural distortion and/or muscle surgery.

Strabismus surgery: Pehere et al. described the management of one patient with binocular diplopia secondary to sickle cell retinopathy associated with ERM formation [9]. Therapeutically, the authors implemented a central mini-tenotomy of the left superior rectus muscle, a targeted surgical approach designed to address small-angle strabismus, combined with environmental modifications aimed at reducing peripheral fusion cues. This dual strategy resulted in successful symptomatic improvement, suggesting that even in cases primarily driven by retinal pathology, modulation of ocular alignment and sensory input can restore functional single vision. The case underscores the importance of individualized, pragmatic management strategies for macular diplopia, particularly in low-resource environments, and supports the role of minimally invasive strabismus procedures as a viable adjunct in select patients [9].

PPV/ERM peeling: PPV with ERM peeling, with or without ILM peeling, remains the standard of care for progressive or symptomatic ERM.

Persistent diplopia may result from cortical adaptation failure despite retinal realignment. Mirzaei et al. found that ERM peeling normalized retinal registration in 80% of patients, but 20% developed new diplopia due to postoperative aniseikonia [26].

Foroozan and Arnold reported a case of diplopia resolution after ERM peeling in a pseudophakic patient, showing that outcomes can vary depending on the chronicity and degree of retinal distortion [19].

A recent innovation is nonvitrectomizing vitreous surgery (NVS), which minimizes retinal trauma while peeling the membrane. Xue et al. have found that NVS produced equivalent anatomical recovery to traditional vitrectomy, with faster recovery and potentially reduced postoperative aniseikonia [28]. Xue et al. analyzed 117 patients (117 eyes) with idiopathic ERM, comparing outcomes between NVS (54 eyes) and PPV (63 eyes) [28]. Baseline characteristics, including best-corrected visual acuity (BCVA), central macular thickness (CMT), and disorganization of the retinal inner layers (DRIL), were comparable between the groups. Postoperatively, both surgical approaches resulted in significant anatomical and functional improvement, with reductions in CMT and DRIL and corresponding gains in BCVA at one and six months. Importantly, no significant differences in structural outcomes were observed between NVS and PPV, supporting the efficacy of NVS as a less invasive alternative [28]. At six months, visual acuity improvement was greater in the NVS group, suggesting a potential functional advantage of preserving vitreous integrity [28]. Additionally, DRIL severity emerged as a prognostic factor, with better visual outcomes in eyes with absent or mild DRIL, although meaningful improvement was still observed in severe cases. Notably, no ERM recurrence was reported in either group during follow-up. These findings indicate that NVS can achieve outcomes comparable to conventional PPV while offering procedural simplification and highlight the importance of retinal microstructural biomarkers, such as DRIL, in predicting postoperative visual recovery [28].

Structured approach: For patients with mechanical or postoperative diplopia, stepwise management, including prisms, buckle removal, and then strabismus surgery, can restore fusion.

Hatt et al. evaluated 50 adult patients (50 patients) with CPR-type diplopia due to retinal misregistration, most commonly associated with ERM (44/50) and less frequently other retinal disorders (6/50) [15]. All patients were assessed using a standardized Diplopia Questionnaire, and multiple treatment modalities were explored, including prisms, Bangerter filters, iseikonic manipulation, and ERM peeling. Overall, 34% (17/50) of patients achieved treatment success, defined as “never” or “rarely” experiencing diplopia at distance and near. These findings highlight the therapeutic challenge of CPR-type diplopia, reflecting its complex sensory origin rather than a purely motor misalignment [15]. Treatment outcomes varied significantly across modalities. Fresnel prism demonstrated the highest success rate (57%, 4/7) among nonsurgical options, whereas Bangerter filters showed limited benefit (14%, 4/28), and iseikonic manipulation was largely ineffective (4%, 1/23). Notably, ERM peeling achieved a relatively high success rate (44%, 8/18), suggesting that addressing the underlying retinal distortion may restore binocular function in selected patients. These results emphasize that while nonsurgical strategies may provide symptomatic relief in some cases, surgical intervention targeting macular pathology can play a meaningful role. Clinicians should adopt an individualized, trial-based approach when managing CPR-type diplopia, given the variability in treatment response [15].

In Langmann et al.'s study, 80% of patients with post-retinal detachment diplopia regained binocular vision after such staged intervention [20]. Langmann et al. evaluated 264 patients (264 eyes undergoing encircling procedure) for retinal detachment to assess the frequency and characteristics of postoperative diplopia due to motility disorders [20]. Management strategies varied, with most patients treated conservatively using prism correction, including prism foils and prism glasses, while a smaller subset required strabismus surgery (three patients) due to persistent deviation, often related to hypertrophic scarring (adhesion syndrome) [20]. One patient required occlusion for intractable diplopia. Importantly, removal of the encircling band did not improve motility, supporting the conclusion that fibrotic changes rather than the implant itself are the primary driver of diplopia. The authors emphasize preventive strategies, including minimizing surgical trauma, early refractive correction, anti-inflammatory therapy, and postoperative motility exercises to reduce the risk of persistent binocular dysfunction [20].

Fison and Chignell evaluated 311 patients (311 eyes undergoing retinal detachment surgery), of whom 15 patients (15 eyes; 5%) developed persistent diplopia lasting more than three months postoperatively [29]. Treatment followed a structured approach beginning with prism correction, which alone resolved diplopia in 40% (6/15) of cases. For patients unresponsive to prisms, removal of the scleral buckle restored single vision in 20% (3/15), suggesting a mechanical contribution to ocular misalignment. In more resistant cases, strabismus surgery, often on the fellow (untreated) eye, provided the most consistent outcomes, either alone or combined with prior interventions. Despite these measures, 20% (3/15) remained symptomatic. These findings emphasize that diplopia after retinal detachment surgery is primarily motor in origin and often treatable, with a stepwise escalation from conservative to surgical management yielding favorable long-term results [29].

Other surgery: Some clinicians employ optical blur therapy using high-plus intraocular lenses (IOLs) or induced defocus in one eye. Ismaiel et al. described complete relief from torsional diplopia in a patient after cataract surgery with a high-plus IOL implant [30]. Ismaiel et al. described one patient (one eye), a 73-year-old man with a one-year history of intractable binocular diplopia and metamorphopsia secondary to DFDS associated with ERM [30]. The patient failed conventional management, including prism correction, and was not a candidate for standard occlusion strategies due to cosmetic concerns and physical limitations. This case highlights the severe functional burden and therapeutic resistance that can characterize retinal misregistration-related diplopia, particularly when both sensory conflict and patient-specific factors limit standard treatment options [30]. As an alternative approach, the patient underwent cataract extraction with implantation of a high-plus IOL to induce extreme monocular blur, effectively suppressing the conflicting retinal image [30]. This strategy successfully eliminated diplopia without the need for external occlusion. The report demonstrates that intentional optical degradation of one eye can serve as a viable solution in refractory cases, particularly when other modalities are not feasible. It also underscores the importance of individualized treatment planning in dragged-fovea diplopia, where functional goals and patient preferences must guide management decisions [30].

Clinical implications and future perspectives

ERM-induced macular diplopia remains a rare but visually disabling complication, particularly in elderly patients or those following cataract surgery. This condition highlights the complex interface between retinal structure and binocular fusion mechanisms.

Recent advances in OCT and OCTA have enabled detailed visualization of microstructural abnormalities, but the persistence of diplopia after anatomical repair suggests the need to address cortical adaptation. Emerging research into neuroplasticity and visual fusion retraining, potentially through virtual reality-based therapies, offers new hope for restoring single vision [18].

Future work should focus on developing standardized diagnostic algorithms that combine microperimetry, retinal registration mapping, and functional imaging, as well as on identifying biomarkers predictive of postsurgical persistence of diplopia [31].

Ultimately, managing macular diplopia requires a multidisciplinary approach, integrating retinal surgery, orthoptic evaluation, and visual neuroscience. Conservative measures remain first-line, while surgical and rehabilitative therapies should be individualized based on the patient’s sensory adaptability and visual demands.

## Conclusions

Macular diplopia secondary to an ERM is an uncommon but visually disabling condition resulting from retinal misregistration and disruption of central fusion. Accurate diagnosis relies on detailed clinical evaluation and multimodal imaging, particularly OCT. While conservative treatments such as prisms and partial occlusion may relieve symptoms, surgical membrane peeling often improves anatomy but not always binocular function. Persistent diplopia highlights the need for considering cortical adaptation in management. A multidisciplinary approach integrating retinal and orthoptic care remains essential to achieve optimal visual outcomes and improve patients’ quality of life.
